# The Implications of Connexin 43 Deficiency during the Early Stages of Chemically Induced Mouse Colon Carcinogenesis

**DOI:** 10.3390/antiox11122368

**Published:** 2022-11-30

**Authors:** Sara Gomes Espírito Santo, Tereza Cristina da Silva, Mathieu Vinken, Bruno Cogliati, Luís Fernando Barbisan, Guilherme Ribeiro Romualdo

**Affiliations:** 1 Department of Pathology, Botucatu Medical School, São Paulo State University (UNESP), Botucatu 18618-687, São Paulo, Brazil; 2 School of Veterinary Medicine and Animal Science, University of São Paulo (USP), São Paulo 05508-270, São Paulo, Brazil; 3 Department of In Vitro Toxicology and Dermato-Cosmetology, Vrije Universiteit Brussel (VUB), 1090 Brussels, Belgium; 4 Department of Structural and Functional Biology, Botucatu Medical School, São Paulo State University (UNESP), Botucatu 18618-689, São Paulo, Brazil

**Keywords:** aberrant crypt foci, colon carcinogenesis, colorectal adenoma, connexin 43, C57BL/6J mouse, 1,2-dimethylhydrazine

## Abstract

Colorectal cancer (CRC), associated with an increased intake of processed red meats, saturated fats, and simple carbohydrates accompanied by low dietary fiber, fruits, and vegetables consumption, presents a high epidemiological burden. Connexin43 (Cx43) protein, which forms gap junctions or hemichannels, has tumor suppressor or oncogenic activities in a cancer type- and stage-dependent manner. Cx43 expression varies during colon carcinogenesis, and its functional role is not fully understood. Thus, we evaluated the implications of Cx43 heterologous deletion (Cx43^+/−^) during the early stages of a chemically induced model of colon carcinogenesis. Female C57BL/6J mice (wild-type or Cx43^+/−^) were submitted to a colon carcinogenesis model induced by 1,2 dimethylhydrazine (DMH). Mice were euthanized eight hours (week 7) or 30 weeks (week 37) after the last DMH administration to evaluate subacute colon toxicity outcomes or the burden of (pre)neoplastic lesions, respectively. At week 7, Cx43 deficiency inferred no alterations in the DMH-induced increase in systemic (peripheral blood), in situ (colonocytes) DNA damage, and apoptosis in the colonocytes. At week 30, Cx43^+/−^ mice presented an increase in preneoplastic aberrant crypt foci (ACF) multiplicity, while no alterations were observed in colorectal adenoma (CRA) occurrence, multiplicity, volume, proliferation, growth, and β-catenin immunoexpression. Similarly, an in silico analysis of human CRA showed decreased mRNA expression of Cx43 with no correlation with proliferation, apoptosis, and β-catenin markers. These findings indicate the discrete role of Cx43 in the early stages of chemically induced mouse colon carcinogenesis.

## 1. Introduction

Colorectal cancer (CRC), which is mainly a sporadic disease (55–70%) associated with inappropriate dietary patterns and lifestyle, accounts for as much as 10% of all incident cases and cancer-related deaths worldwide, totaling about 2 million new cases and nearly 1 million deaths annually [1]. By 2040, there will be 63–67% and 77–80% projected increases in incidence and mortality, respectively [1]. The current CRC epidemiological burden is closely related to the human development index (HDI), as, in the high HDI Western countries, the efficient diagnosis and screening methods are responsible for the decreased incidence and increased survival. In middle-to-low HDI countries, CRC screening is quite heterogeneous, leading to late diagnosis (~25% cases), which is usually associated with advanced disease stage, synchronous metastases, and reduced survival rates (up to ~10%) [2,3,4]. In these countries, the so-called “Westernization of lifestyle”, which includes the use of tobacco and alcoholic beverages, physical inactivity, increased intake of processed red meats, low dietary fiber, fruits, and vegetables, accounts for the increased CRC risk and burden [5,6,7,8,9].

The complex and multistage process in which sporadic CRC emerges, defined as colorectal carcinogenesis, encompasses a morphological–molecular sequence based on the transformation of the normal colorectal glandular epithelium into a colorectal adenoma (CRA) and, ultimately, into an invasive and metastatic adenocarcinoma [10,11]. Over the past decades, several attempts have been made to enable the early diagnosis of CRC, and the detection of aberrant crypt foci (ACF) as preneoplastic lesions has been long postulated in both human and rodent studies [12,13,14,15]. ACF consists of methylene blue stainable, large, “slit-like”, and thick aberrant crypts (AC), featuring KRAS mutations in humans and p53 or β-catenin mutations and accumulation in rodents, alterations that also feature in the late stages of colon carcinogenesis [16,17,18]. Accumulating clinical, morphological, and molecular evidence indicates that ACF are precursors of CRA and, ultimately, CRC [19]. In particular, short-to-medium-term chemically induced bioassays in both rats and mice have been widely applied in preclinical research to unveil the potential predisposing of promoting or preventive agents, as well as the screening of putative molecular targets. Among those, 1,2-dimethylhydrazine (DMH) and its metabolite azoxymethane (AOM) are organ-specific complete carcinogens capable of inducing ACF and neoplastic lesions mainly located in the middle and distal colon of different rat and mouse strains [20,21,22,23]. In the liver of rodents, DMH is oxidized to AOM—that is, hydrolyzed to methylazoxymethanol (MAM). MAM reaches the colon through bile or blood circulation, and it is converted into alkylating reactive metabolites by β-glucuronidase, β-glucosidase, and sulfatase bacterial enzymes [24,25,26]. In brief, these molecules implicate oxidative stress and genomic instability—which is a cancer hallmark—in the colonocytes, leading to the emergence and accumulation of epigenomic or genomic alterations in key oncogenes or oncosuppressor genes. In fact, not only the oxidative stress but also the antioxidant status (e.g., enzymatic and nonenzymatic antioxidant agents) are directly implicated in different stages of rodent and human colon carcinogenesis [20,27]. These molecular alterations reflect on the appearance of ACF, which may progress to colorectal neoplasia [19,28].

Connexins are part of a family of proteins composed of 21 isoforms classified according to their molecular weight. These proteins are integral membrane proteins that may form functional hemichannels, enabling the communication between the cytosol and extracellular milieu, which may further dock between adjacent cells to form gap junctions, which facilitate intercellular communication. Both hemichannels and gap junctional intercellular communication (GJIC) enable the bidirectional exchange of small and hydrophilic molecules (e.g., microRNAs, metabolites, ions, and secondary messengers) playing essential roles in cell growth, differentiation, and tissue homeostasis. Furthermore, channel-independent biological functions have also been described for connexin proteins, as such [29,30,31,32]. Of all connexins, Cx43 is most ubiquitously expressed in the normal gastrointestinal system, including the colon, where it is sparsely distributed in intracellular compartments, and punctuated in the plasma membrane, likely representing GJIC in the colonic mucosa [33]. 

During colon carcinogenesis, the roles of Cx43 as a tumor suppressor or oncogene are not fully understood [34]. Although the majority of CRC are positive for the Cx43 protein, findings suggest that Cx43 may be absent/downregulated in CRC samples and cell tumor lines, which is associated with a loss of GJIC function. Indeed, the absence of Cx43 expression (~10% CRC) goes hand in hand with poor clinical outcomes, such as advanced stage, the presence of metastasis, and reduced relapse-free and overall survival [35,36,37]. In a cohort of 29 human microdissected samples of benign adenomatous polyps to poorly differentiated CRC, only Cx43 was found to be mutated in the multifunctional C-terminal domain of the protein, and its expression was restricted to invasive structures of exophytic CRC (50%) while absent in CRA and endophytic CRC, thereby demonstrating a potential role of the mutated Cx43 protein in the advanced stages of colon carcinogenesis [38]. These clinical findings indicate that the maintenance of nonmutated Cx43 expression and its normal function may act as a tumor suppressor during colon carcinogenesis. In this respect, the transfection of Cx43 into human CRC HT29 cells, which do not express the Cx43 protein, reduced the growth of these cells in monolayer and tumor xenografts by partly colocalizing with β-catenin and downregulating the Wnt signaling pathway, which is frequently activated in colon carcinogenesis [35]. In contrast to these findings, in a cohort of 50 samples, Cx43 immunoexpression was found to progressively increase from CRA to the late stages of CRC, although no association with the clinical parameters was established [39]. More recently, the transfection of Cx43 into HCT116 and HT-29 spheroids was found to upregulate mTOR, downregulate AMPK signaling, and increase the ATP content and glucose oxygen consumption in a GJIC-dependent manner, leading to a growth advantage in spheroids and xenografts [40]. In addition, the analysis of The Cancer Genome Atlas (TCGA) database indicated that higher Cx43 levels are associated with worse clinical outcomes in CRC (e.g., advanced stage, presence of lymph metastasis, and invasion depth) [40]. 

Although Cx43 is downregulated or displays aberrant localization in human CRA [41], the available experimental and clinical data predominantly encompass the late stages of colon carcinogenesis, and early stages are usually underappreciated. For this reason, the present study evaluated the implications of Cx43 heterologous deletion (Cx43^+/−^) in mice on the early steps of chemically induced colon carcinogenesis. This is the first report on the use of Cx43-deficient animals on colon carcinogenesis. These findings show a minor role of this protein on the subacute carcinogen-induced outcomes and also reveal a discrete effect on preneoplastic and neoplastic lesion development.

## 2. Materials and Methods

### 2.1. Animals

Female wild-type (WT) C57BL/6J mice were obtained from the School of Veterinary Medicine and Animal Science of the University of São Paulo (USP, São Paulo—SP, Brazil) and kept in Botucatu Medical School of the São Paulo State University (UNESP, São Paulo—SP, Brazil). Heterozygous Cx43-deficient mice (Cx43^+/−^) were generated by replacing exon 2 of the Cx43 gene with the neomycin resistance gene and backcrossed with WT C57BL/6J. Heterozygous animals were used, since the corresponding homozygous null mutation for Cx43 is lethal [42].

### 2.2. Experimental Design and Sampling

**Figure 1:**
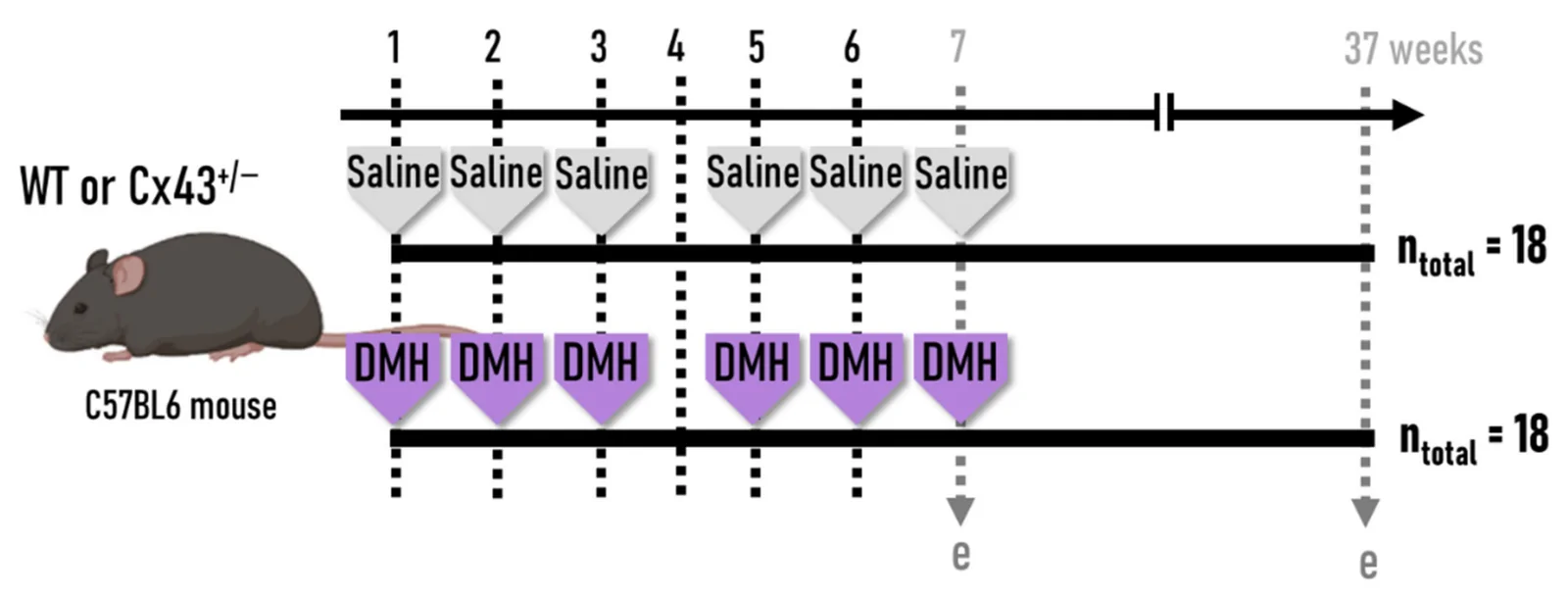
Experimental design. Saline: 1×NaCl 0.9% i.p. administration; DMH: 1 × 20 mg/kg/b.wt. i.p. administration; e: euthanasia; n_total_: total number of mice per genotype.

After a 2-week acclimation period, six-week-old female WT and Cx43^+/−^ C57BL/6J mice were subjected to the well-established 1,2 dimethylhydrazine (DMH)-induced model of colon carcinogenesis. Animals were subjected to 2 cycles of DMH injection (20 mg/kg body weight (b.wt.)) or NaCl% 0.9% vehicle intraperitoneal (i.p.) injections once a week for three weeks (weeks 1–3 and 5–7), with a week of rest between them (week 4) (*n* = 18 animals/genotype) (Figure 1). The dose of DMH carcinogen and the experimental design were based on the findings of Richards [43] and James et al. [44]. Both vehicle and DMH-injected animals (*n* = 6 mice/genotype) were euthanized at an early timepoint (week 7), namely 8 h after the last DMH administration, to evaluate the effects of Cx43 on carcinogen-induced genotoxicity and subacute colon toxicity outcomes. This timepoint comprises the colonic crypt apoptosis peak after DMH injection [45]. The remaining animals (*n* = 12 mice/genotype) were euthanized at a late timepoint (week 37), namely 30 weeks after the last DMH administration, aiming for the screening of (pre)neoplastic lesions (Figure 1). Both euthanasia procedures were performed by exsanguination under ketamine/xylazine anesthesia (100/16 mg/kg b.wt., i.p.). The tail end was collected for Cx43 genotyping, and the liver was collected and weighted. The animals received chow and water ad libitum. The animals were housed in polypropylene cages (4 animals/cage) in a room maintained at 22 ± 2 °C, 55 ± 10% humidity and with a 12-h light/dark cycle (light between 07:00 a.m. and 07:00 p.m.). 

At both timepoints (weeks 7 and 37), the large intestine was removed, opened longitudinally, and gently rinsed with 0.9% saline to remove residual bowel contents, and the length was then measured (cm). At week 7, distal colon samples (1–2 cm) were collected, snap-frozen in liquid nitrogen, and stored at −80 °C for biochemical analysis. Subsequently, the colon specimens were fixed flat in methacarn solution (60% methanol, 30% chloroform, and 10% acetic acid) for 12 h (4 °C) and transferred to 70% alcohol. At week 37, methacarn-fixed colons were stained with 1% methylene blue solution for analysis and the quantification of preneoplastic ACF [12]. At this timepoint, macroscopic tumors were counted, measured with a digital caliper, excised from the colon specimens, and individually fixed in methacarn. At both weeks 7 and 37, flat-fixed distal colon specimens (~2 cm width segments) and tumors were embedded in paraffin for histological analysis. Distal colon specimens were collected at both timepoints, since the increased amount of DMH-induced DNA adducts in this section was positively correlated to the higher incidence of (pre)neoplastic alterations compared to the middle/proximal colon sections [44,46]. All procedures described were approved by the Ethics Committee on Animal Use of the São Paulo State University (UNESP) (acceptance number 569210721).

### 2.3. Genotyping

DNA was extracted from the tail end of mice, and a polymerase chain reaction (PCR) was performed using primer pairs oIMR0092 (AAT CCA TCT TGT TCA ATG GCC GAT C), oIMR6174 (AGG TGG TGT CCA GAG CCT TA), and oIMR6175 (CAC GTG AGC CAA GTA CAG GA) (Jackson Laboratory, Bar Harbor, ME, USA). The cycling conditions were: 1 cycle (94 °C for 2 min.); 10 cycles (94 °C for 20 s, 65 °C for 30 s, and 68 °C for 40 s); 28 cycles (94 °C for 20 s, 60 °C for 35 s, and 72 °C for 40 s); and 1 cycle (72 °C for 5 min). Samples were loaded onto a 2% agarose gel with a specific nucleic acid dye at 80 V for 45 min. The PCR product was evaluated in the Chemidoc image system (Bio-Rad Laboratories, Hercules, CA, USA). Representative gel photos can be observed in Supplementary Figure S1. 

### 2.4. Genotoxicity Assessment

Peripheral blood samples from the orbital plexus were obtained 4h after the last DMH administration (week 7) for the alkaline single-cell gel electrophoresis assay. The timepoint was based on a similar approach that identified increased colon genotoxicity after a single DMH injection at the same dose (and route) [47]. Blood samples were mixed with low-melting point agarose (100 μL 0.75% in PBS, Invitrogen, Thermo Fisher Scientific, Waltham, MA, USA), spread on slides precoated with normal point agarose (1.5% in PBS, Invitrogen), and coverslipped. Following agarose solidification (4 °C for 5 min), coverslips were carefully removed, and slides were incubated with cold lysis solution (2.5 M NaCl, 100 mM Na_2_EDTA, 10 mM Tris–HCl, 1% Triton X-100, and 10% DMSO, pH 10) overnight at 4 °C. Subsequently, the slides were immersed in fresh cold alkaline electrophoresis buffer (300 mM NaOH, 1 mM Na_2_EDTA, pH > 13) for 20 min. Electrophoresis was conducted for 20 min at 1 V/cm (300 mA). The slides were neutralized with 0.4 M Tris (pH 7.5), dehydrated in 100% ethanol, and stained with SYBR Gold solution (1:10,000) (Thermo Fisher Scientific, Waltham, MA, USA). Fifty randomly selected nucleoids were counted per slide (duplicate) in an epi-fluorescence microscope (Olympus BX-50, Olympus Corporation, Tokyo, Japan) using Comet Assay IV software (Perceptive Instruments, Bury St Edmunds, UK). The tail intensity (% of DNA in the comet tail) was calculated. 

### 2.5. ACF Screening and Tumor Analysis

In methylene blue-stained colon specimens, ACF number/cm (mean number of ACF per colon length), aberrant crypt (AC)/cm (mean number of AC per colon length), and ACF multiplicity (mean number of AC per ACF) were calculated for each group [12]. During necropsy, macroscopic gross tumors were measured using a digital caliper, and the tumor volume was calculated using the following formula: length × width × depth × 1/2. Sections (5 μm) were obtained from methacarn-fixed and paraffin-embedded tumor samples and stained with hematoxylin and eosin (HE). In HE-stained sections, neoplastic lesions were identified and further classified into CRAs (differential features: polypoid lesions with branching hyperplasic/dysplastic tubular crypts and the reduction/absence of goblet cells restricted to the mucosa), according to the “International Harmonization of Nomenclature and Diagnostic Criteria for Lesions in Rats and Mice” criteria [48]. The tumor incidence (percentage of CRA-bearing mice) and multiplicity (mean number of CRA per mouse) were calculated. 

### 2.6. Immunohistochemistry Analysis

Tumor or distal colon sections (5 µm) were subjected to antigen retrieval in 0.01 M citrate buffer (pH 6.0, 5 min, 120 °C) in a Pascal Pressure Chamber (Dako Cytomation, Denmark). Following an endogenous peroxidase blockade with 10% H_2_O_2_ in phosphate-buffered saline (PBS) (15 min), the slides were treated with skim milk (60 min) and incubated with primary antibodies directed against Ki-67 (i.e., cell proliferation, MA5-14520, 1:100 dilution, Thermo Fisher Scientific, Waltham, MA, USA), cleaved caspase-3 (i.e., apoptosis, PA577887, 1:50 dilution, Thermo Fisher Scientific, Waltham, MA, USA), phosphorylated-H2AX (i.e., DNA damage marker, MA5-27753, 1:200, Thermo Fisher Scientific, Waltham, MA, USA), or β-catenin (ab32572, E247, 1:400 dilution, Abcam, Cambridge, UK) in a humidified chamber (overnight, 4 °C). The slides were incubated with a one-step horseradish peroxidase polymer (EasyPath—Erviegas, Indaiatuba, São Paulo, Brazil) (20 min). The reaction was visualized with 3-diaminobenzidine (DAB) chromogen (Sigma Aldrich, Burlington, MA, USA) and counterstained with Harris’ hematoxylin. A semiquantitative analysis for Ki-67, cleaved caspase-3, and H2AX was performed in 15 randomly selected crypts/animal/section (distal colon) or 6 fields (CRA) (20× objective, 1 section/animal). Both positive and negative epithelial cells were counted, and the results were expressed as the percentage (%) of Ki-67+, cleaved caspase-3+, or H2AX+ cells per crypt or CRAs. The growth index of each tumor was also calculated, considering the % of proliferating cells (Ki-67+) minus the % of apoptotic cells (caspase-3+). For β-catenin, the occurrence (%) of each pattern of staining (nucleus, cytoplasm, and/or membrane) was evaluated in all CRA. A score of intensity was also calculated for this marker, regardless of cellular localization: 0 (absence), 1 (weak), 2 (moderate), and 3 (strong). The analyses were performed by using ImageJ software (National Institutes of Health, Bethesda, MD, USA).

### 2.7. Analysis of Antioxidant Enzymes

Total proteins were extracted from 50–100 mg of distal colon samples in KCl buffer (1.15%) and were quantified by Bradford’s method. Total proteins were used to determine the activities of superoxide dismutase (SOD) [49] and catalase [50]. SOD activity was measured based on the inhibition of the superoxide radical reaction with pyrogallol. The catalase activity was determined in a 30% H_2_O_2_ PBS solution. The absorbance was measured at 420 (SOD) or 240 (catalase) nm in a microplate reader (Spectra Max 190, Molecular Devices, Silicon Valley, CA, USA).

### 2.8. In Silico Analysis of Human CRA Samples

Using the R2: Genomics Analysis and Visualization Platform (https://r2.amc.nl/, accessed on 1 June 2021), the mRNA levels of GJA1 (Cx43, Gene ID: 2697) and its correlation with β-catenin (CTNNB1, gene ID: 1499), caspase-3 (CASP3, gene ID: 836), Ki-67 (MKI67, gene ID: 4288), and other classical colon carcinogenesis-related genes (Supplementary Figure S2 and Figure S3) were evaluated. The analysis was performed in two different publicly available datasets: Galamb—18—MAS5.0—u133p2 (GEO ID: gse15960) dataset [51], which comprised a whole-genome oligonucleotide microarray analysis of laser microdissected human normal mucosa, CRA, and CRC samples (*n* = 6 for each class), and Balazs—53—MAS5.0—u133p2 (GEO ID: gse4183) dataset [52], which was also selected and comprised high-density oligonucleotide microarray data of whole biopsy samples of the normal colon (*n* = 8), CRA (*n* = 15), and CRC (*n* = 15) samples.

### 2.9. Statistical Analysis

Biological correlations were performed by using Pearson’s correlation test (Pearson’s r coefficient). CRA incidence data were analyzed by Fisher’s exact test. Pairwise comparisons were evaluated by Student’s *t*- or Mann–Whitney tests. Other data were analyzed using the one-way analysis of variance (ANOVA) or Kruskal–Wallis tests, followed by a *post hoc* Tukey test. Data presentation and the number of replicates are detailed in the footnotes and captions. The aforementioned analyses were performed using Prism GraphPad software (V4.03, GraphPad Software, San Diego, CA, USA). The level of significance was set at *p* < 0.05. 

## 3. Results

### 3.1. Cx43 Deficiency Does Not Alter DMH Effects on Body Weight, Liver Weights, or Colon Length at Weeks 7 and 37

**Figure 2:**
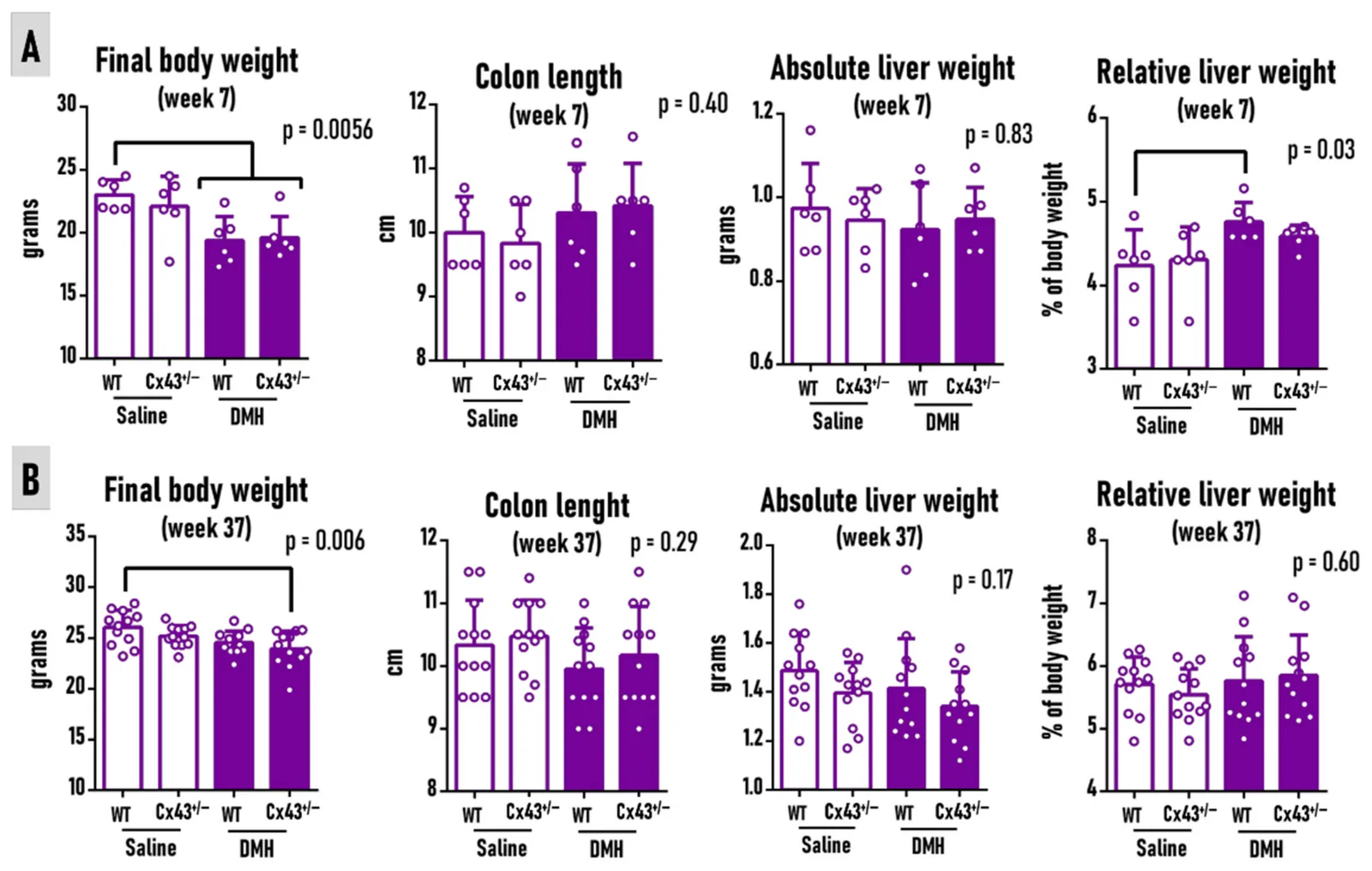
Implications of connexin 43 heterologous deletion (Cx43^+/−^) on the final body weight, colon length, and absolute and relative liver weights during 1,2-dimethylhidrazine (DMH)-induced colon carcinogenesis in C57BL/6J mice at weeks (**A**) 7 and (**B**) 37. WT: wild-type. *n* = 6 (week 7) or 12 (week 37) mice/group/genotype. Data were presented as mean + standard deviation and data points. Data were analyzed by the one-way ANOVA and *post hoc* Tukey test (*p* < 0.05).

At week 7, eight hours after the last carcinogen administration, animals that received six applications of DMH showed decreased body weights compared to saline-treated WT animals (*p* = 0.0056), regardless of the genotype (Figure 2A). The colon length and absolute liver weight were similar between both genotypes, independent from carcinogen administration (Figure 2A). DMH-administered WT mice had increased relative liver weights compared to their WT saline counterparts (*p* = 0.03) (Figure 2A). At week 37, only DMH-treated Cx43^+/−^ mice displayed decreased body weights compared to the saline-treated WT animals (*p* = 0.006) (Figure 2B). Nonetheless, the colon length and liver weights remained unmodified in both genotypes, regardless of carcinogen administration (Figure 2B). At both moments analyzed, the food intake remained unchanged among groups (data not shown).

### 3.2. Cx43 Deficiency Does Not Alter Carcinogen-Induced Outcomes at Week 7

**Figure 3:**
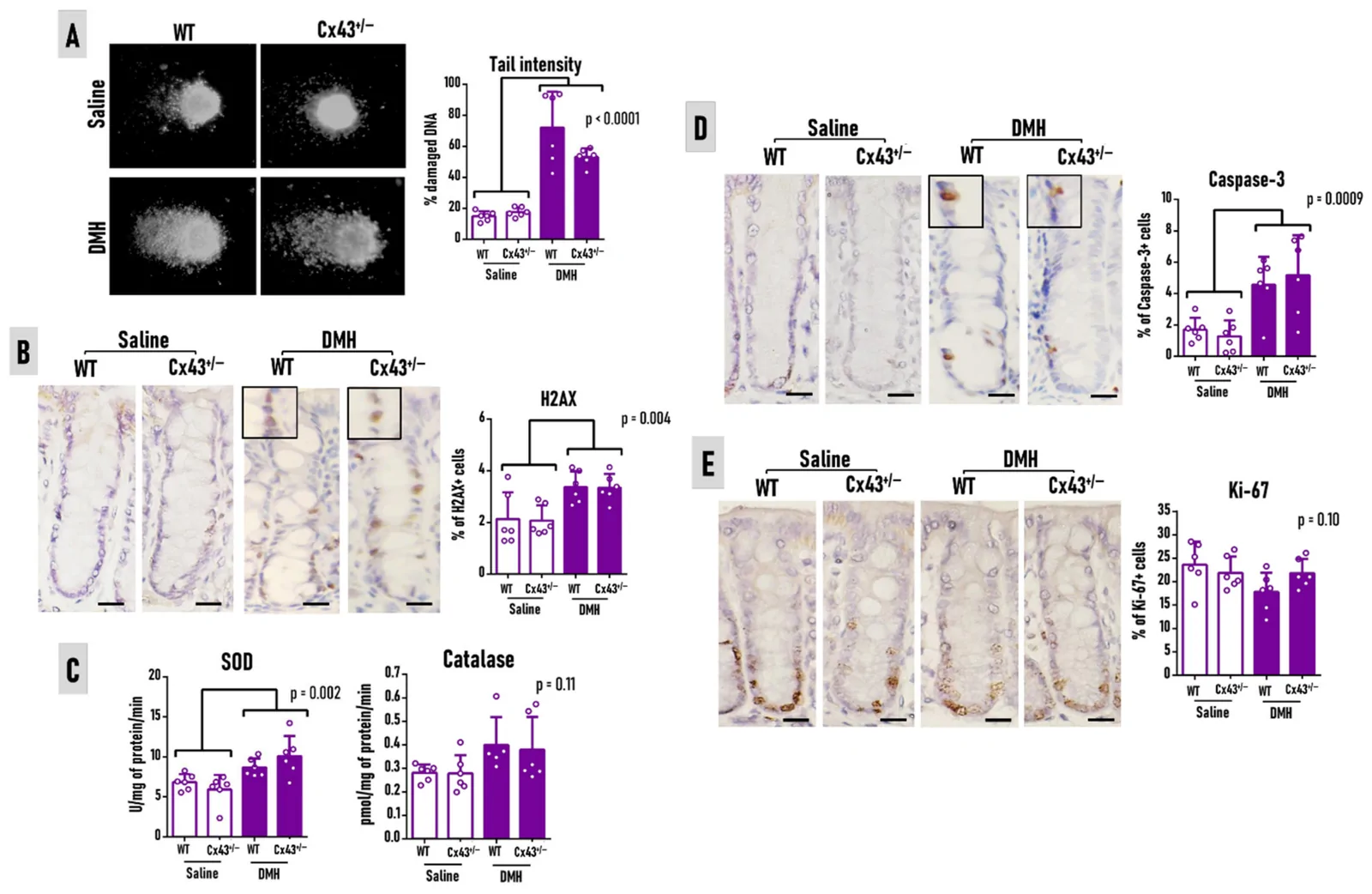
Implications of connexin 43 heterologous deletion (Cx43^+/−^) on (**A**) peripheral blood genotoxicity, (**B**) colonocyte genotoxicity (H2AX), (**C**) antioxidant superoxide dismutase (SOD) and catalase enzymes, (**D**) epithelial crypt apoptosis (caspase-3), and (**E**) epithelial crypt proliferation (Ki-67) during 1,2-dimethylhydrazine (DMH)-induced colon carcinogenesis in C57BL/6J mice at week 7. (**A**) Representative nucleoids of the comet assay are presented. (**B**,**D**,**E**) A representative microscopic overview (scale bar: 20 µm) and semiquantitative analysis of H2AX, Ki-67, and cleaved caspase-3 immunostaining in the colonocytes in both genotypes are presented. WT: wild-type. *n* = 6 mice/group/genotype. Data were presented as mean + standard deviation and data points. Data were analyzed by the one-way ANOVA and *post hoc* Tukey test (*p* < 0.05).

Eight hours after the last carcinogen administration, the Cx43^+/−^ genotype did not modify the DMH-mediated increase in DNA damage in leukocytes from peripheral blood (comet assay) (*p* < 0.0001) (Figure 3A) or in colonocytes (H2AX expression) (*p* = 0.004) (Figure 3B). The SOD activity was significantly increased (*p* = 0.002), while catalase showed a trend of enhancement in response to DMH exposure, independent from the genotype (Figure 3C). Furthermore, the Cx43 deficiency did not alter the increase in colonocyte apoptosis (caspase-3) caused by genotoxicity induced by DMH (*p* = 0.0009) (Figure 3D). Both groups showed similar colonocyte proliferation levels, regardless of carcinogen administration or Cx43 deficiency (*p* = 0.10) (Figure 3E). Collectively, the results indicate that Cx43 did not alter the subacute carcinogen effects on the colonic mucosa.

### 3.3. Cx43 Deficiency Slightly Attenuates Preneoplastic ACF Burden at Week 37

**Figure 4:**
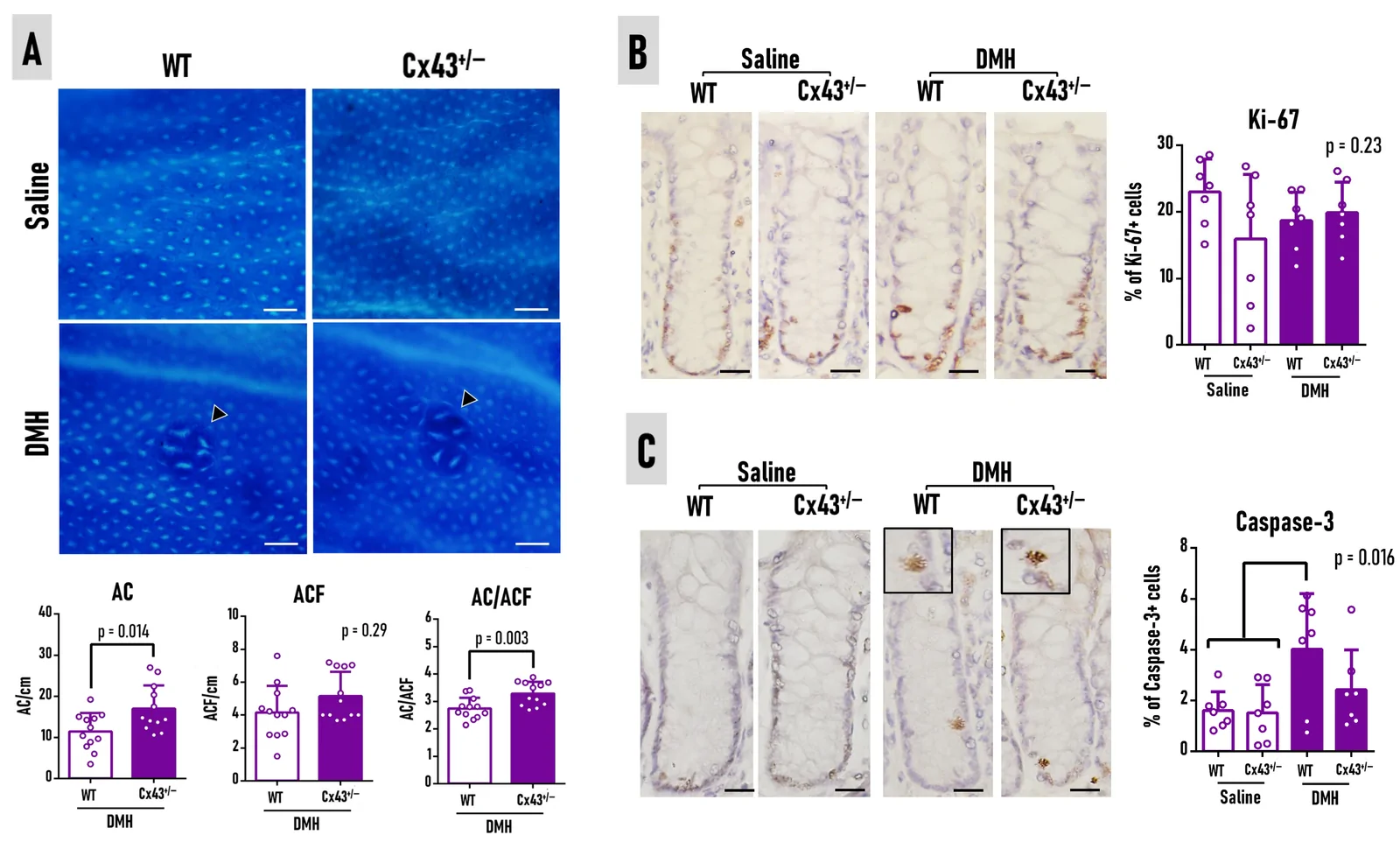
The implications of connexin 43 heterologous deletion (Cx43^+/−^) on (**A**) the aberrant crypt foci (ACF) burden, (**B**) epithelial crypt proliferation (Ki-67), and (**C**) apoptosis (caspase-3) during 1,2-dimethylhydrazine (DMH)-induced colon carcinogenesis in C57BL/6J mice at week 37. (**A**) Representative photomicrographs of a whole mount of colon specimens stained with methylene blue (scale bar: 100 µm) depicting the absence (noninitiated) or the occurrence of ACF (DMH-initiated mouse) (arrowheads) and colon length, ACF, AC, and AC multiplicity (AC/ACF) data in both genotypes. (**B**) Representative microscopic overview (scale bar: 20 µm) and semiquantitative analysis of Ki-67 and cleaved caspase-3 (details) immunostaining in the colonocytes of normal-appearing crypts in both genotypes. WT: wild-type. *n* = 7 (immunohistochemistry) or 12 (ACF data) mice/group/genotype. Data were presented as mean + standard deviation and data points. Data were analyzed by the one-way ANOVA and *post hoc* Tukey test (proliferation and apoptosis) or the Student’s *t*-test (ACF data) (*p* < 0.05).

All DMH-initiated mice in both genotypes developed ACF, which are larger, darker, and thicker than normal crypts, as pictured in the topographic views of whole-colon mounts stained with methylene blue (Figure 4A). The Cx43^+/−^ genotype did not influence the mean number of ACFs but enhanced the average number of ACs (*p* = 0.014) and slightly increased by 17% the ACF multiplicity (AC per ACF) (*p* = 0.003) (Figure 4A). The different genotypes did not alter the colonocyte proliferation (Ki-67) in the “normal-appearing” crypts (Figure 4B). WT mice subjected to the DMH protocol had increased apoptosis in the “normal-appearing” crypts compared to the noninitiated animals (both WT and Cx43^+/−^), an effect that was not observed during Cx43 deficiency (*p* = 0.014) (Figure 4C). Thus, the findings point to a discrete role of Cx43 on crypt cell dynamics and preneoplastic ACF development. 

### 3.4. Cx43 Deficiency Does Not Alter Colorectal Adenoma Burden at Week 37

**Figure 5:**
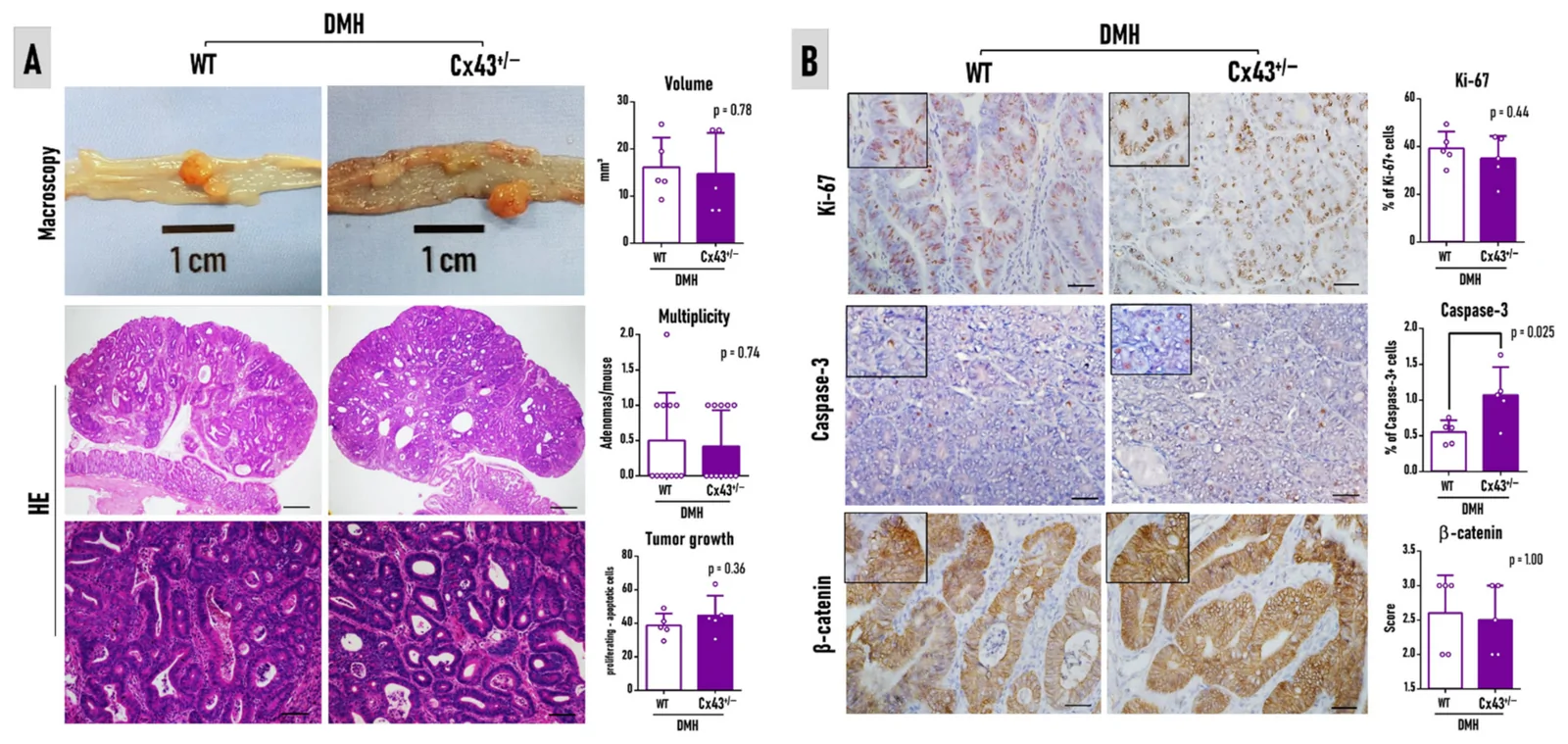
Implications of Cx43^+/−^ on colorectal adenoma (CRA) development during 1,2-dimethylhydrazine (DMH)-induced colon carcinogenesis in C57BL/6J mice at week 37. (**A**) Representative macroscopic (top) and microscopic overview (HE-stained sections, middle (scale bar: 100 µm) and bottom (scale bar: 50 µm)), volume, multiplicity, and tumor growth data of DMH-induced CRAs in both genotypes. (**B**) Representative microscopic overview (details, scale bar: 20 µm) and semiquantitative analysis of Ki-67, cleaved caspase-3, and β-catenin immunostaining of DMH-induced CRAs in both genotypes. WT: wild-type. Data were presented as median + interquartile range (β-catenin) or mean + standard deviation (rest) and data points. Data were analyzed by the Mann–Whitney (β-catenin) or Student’s *t-*test (rest) (*p* < 0.05).

Although the 37-week-long protocol led to a reduced number of gross macroscopic alterations in DMH-treated mice (Figure 5A), all lesions were identified as CRAs after histopathological analysis in HE-stained sections (Figure 5A). Cx43^+/−^ mice showed similar CRA incidence (5/12, 41.6%, for both genotypes), multiplicity, and volume compared to WT animals (Figure 5A). The immunohistochemistry analysis of Ki-67 also revealed a similar proliferation index and β-catenin score in CRAs for both genotypes (Figure 5B). Regarding the cellular localization, all tumors for both genotypes (5/5, 100%) displayed membranous and cytoplasmic β-catenin staining, revealing no nuclear translocation. Although Cx43^+/−^ increased the number of apoptotic cells in CRAs (*p* = 0.025) (Figure 5A), the reduced percentage of apoptotic cells in all tumors (maximum of ~1.5%) did not confer a difference in the tumor growth index (Figure 5B). These results indicate a weak role of Cx43 on CRA development in an early DMH-induced mouse colon carcinogenesis model.

### 3.5. Cx43 mRNA Is Not Correlated to Ki-67, Caspase-3, and β-Catenin in Human Colorectal Adenoma

**Figure 6:**
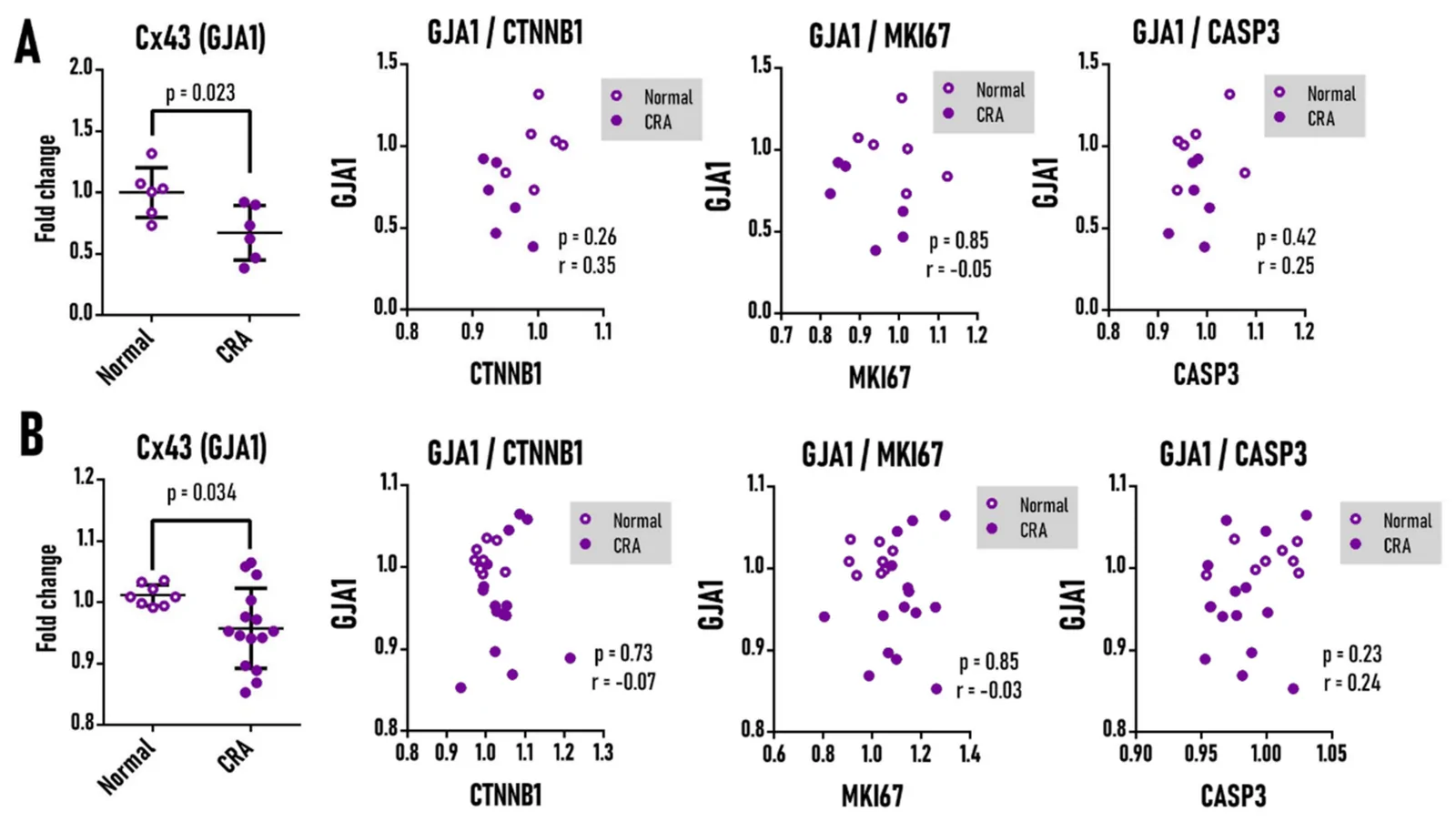
In silico analysis of the Cx43 gene (GJA1) and its correlation with the β-catenin (CTNNB1), Ki-67 (MKI67), and caspase-3 (CASP3) mRNA levels in colonic human samples (**A**) laser microdissected or (**B**) whole biopsy samples of a normal mucosa/colon and colorectal adenoma (CRA). A: human normal mucosa and CRA samples (*n* = 6 for each class); B: normal colon (*n* = 8) and CRA (*n* = 15) samples. Data were presented as mean ± standard deviation and data points. Data were analyzed by the Student’s *t*-test or Pearson’s correlation test (*p* < 0.05).

Both laser microdissected (*p* = 0.023) and whole biopsy (*p* = 0.034) human samples showed that the expression of the GJA1 gene is significantly downregulated in CRA compared to a normal colon (Figure 6). Nonetheless, in line with the preclinical findings, this decreased expression was not correlated to Ki-67, β-catenin, and caspase-3 genes in this benign and polypoid lesion. Furthermore, TP53, another classical gene involved in late CRC development, also showed the absence of correlation with GJA1 expression in CRA (Supplementary Figure S2). Although a significant positive correlation between GJA1 and KRAS in whole biopsy samples of CRA (and not in laser microdissected ones) (Supplementary Figure S2) was established, GJA1 expression was found to be downregulated, and the RAS pathway is usually activated in colon carcinogenesis [53]. In contrast to these findings, GJA1 expression was upregulated in the whole biopsy samples of CRC when compared to both normal colon and CRA (*p* < 0.0001) and was positively correlated with cell proliferation marker MKI67 (*p* = 0.04, r = 0.42) (Supplementary Figure S3).

## 4. Discussion

This study was set to investigate the effects of Cx43 deficiency by using heterologous deletion in female C57BL/6J mice during the early stages of DMH-induced colon carcinogenesis. For this purpose, it was investigated whether Cx43 deficiency would modify (A) the subacute toxicity outcomes caused by DMH (8 h after the last administration) and/or (B) the earliest endpoint colonic lesions induced by this carcinogen (30 weeks after the last administration). Cx43 deficiency inferred no alterations in the DMH-induced increase in DNA damage in leukocytes from the peripheral blood or in colonocytes (H2AX marker), antioxidant enzyme activity, and apoptosis in the colonocytes (caspase-3), which are known subacute carcinogen outcomes [20,21]. Furthermore, although Cx43^+/−^ had increased preneoplastic ACF multiplicity, no alterations were observed in the CRA size, occurrence, multiplicity, growth, proliferation (Ki67), and β-catenin expression. The in silico analysis of human CRA showed decreased mRNA expression of Cx43, yet, in line with the in vivo findings, this downregulation showed no correlation with the proliferation (MKI67) and apoptosis (CASP3) markers, β-catenin, and other classically altered genes during colorectal carcinogenesis. These findings may indicate a discrete implication of Cx43 on the early stages of human and mouse colon carcinogenesis. 

Cx43-deficient mice have proven to be valuable in investigating the participation of Cx43 in gastrointestinal physiology and pathology. Since homozygous Cx43^−/−^ mice die at birth, only heterozygous animals can be used for research purposes. Using Cx43^+/−^ mice, Guttman et al. [54] showed that Cx43 is implicated in the physiology of the colon, as Cx43 hemichannels mediate water release from colonocytes during bacterial infections. Nonetheless, in uninfected mice, Cx43 deficiency alone had no effects on the luminal water content. The findings of the present study corroborate with this, as, in saline-injected mice, Cx43 deficiency did not alter the colon crypt proliferation and apoptosis at both timepoints and did not trigger DNA damage in the peripheral blood and the colon, indicating that Cx43 deficiency per se may not impair the colon crypt dynamics and implicate genotoxicity in general. Biomedical preclinical research in general suffers from a clear male predominance, and models using female animals are usually underappreciated [55]. Despite presenting a slightly lower CRC epidemiological burden that is majorly attributed to healthier lifestyle choices, women are still highly affected by this disease [1]. The indirect tumor-promoting effects of testosterone observed in an AOM-induced model in a C57BL/6J sub-strain, which may also explain the sex disparity in humans [56], should not discourage the use of female mice in chemically induced CRC models reflecting the human disease. 

After administration, the procarcinogen DMH undergoes biotransformation in the liver by cytochrome (CYP) P450 enzymes, mainly by the subunit CYP2E1, and it is metabolized to AOM; MAM; and, ultimately, to the methyl diazonium, methyl ions, and other DNA-reactive species [21,22,23,24,25,26]. These are highly reactive molecules that reach the colon via bile or the bloodstream, alkylating the DNA of colonocytes and contributing to DNA damage and genomic instability cancer hallmarks [21,22,23,24,25,26]. Eight hours after the last DMH dose, increased phosphorylated H2AX immunoexpression was observed in colonocytes. Phospho-H2AX is a sensitive molecular marker of double-stranded breaks (DBS) playing a role in signaling and initiating the repair of these DNA lesions [57]. In agreement with this finding, increased single-stranded breaks (SSB) and DSB in peripheral blood were noticed, as depicted by the comet assay, indicating the systemic bioavailability of DMH and its metabolites [21]. In the colonic mucosa, while some of these alterations are repaired, unfixed DNA adducts and DSB may impair the maintenance of genomic stability, “initiating” colonocytes for the carcinogenesis process. Furthermore, in response to the alkylating carcinogen challenge, to keep mucosal homeostasis, morphological and molecular events switch towards cell cycle arrest and apoptotic events, including an increase in mRNA and protein expression of effector caspases [20,58], as equally observed in the present study. The DMH-induced increase in antioxidant enzymes may indicate that endogen antioxidant systems are acting in response to reactive metabolites generated by carcinogen metabolism 8 h after the last exposure. SOD and catalase are fundamental enzymatic antioxidant agents that act as a first line of defense against carcinogen-induced oxidative stress. SOD catalyzes the dismutation of two molecules of superoxide anion to hydrogen peroxide (H_2_O_2_) and molecular oxygen, while the catalase-mediated degradation or reduction of H_2_O_2_ to water and molecular oxygen completed the detoxification process initiated by SOD [59]. After 24 h, the DMH-induced stress progressively impairs these enzymatic antioxidant systems at the transcriptomic and functional levels in both the liver and colon [20]. Although Cx43-related functions are potentially implicated in promoting drug delivery [59], controlling apoptosis, proliferation [40,60,61,62], and modulating oxidative stress [63,64] in different cell types/organs and biological contexts, no implications of Cx43 deficiency on the DMH-induced subacute challenge outcomes was found in this study. 

The screening of DMH endpoint lesions revealed that Cx43 deficiency increased the number of AC and ACF multiplicity, which could indicate a potential tumor-suppressive role of this protein, as previously addressed in vitro and in a xenograft mouse model using HT29 CRC cells [35]. CRA incidence, multiplicity, proliferation, apoptosis, and β-catenin expression remained unaltered. Rodent data indicate that ACF occurrence is positively correlated with CRA emergence, indicating that the former are potential precursors of the latter [19,28]. However, it is important to point out that ACF emergence is quite dynamic, and only a minor part of ACF progress to CRA. Indeed, ACF regression over time is a phenomenon observed in both mice [65] and humans [66]. Therefore, the increase in ACF burden in Cx43 deficiency should be considered with caution. In the present study, ACF were the main endpoint lesions, and longer studies with an increased CRA burden and CRC development should be further considered.

The tumor suppressor role attributed to Cx43 during colon carcinogenesis is mostly related to the potential impairment of Wnt/β-catenin signaling activation [35]. Ectopically expressed Cx43 in HT29 CRC cells, which are naturally low/absent in Cx43, was found to bind and form a membranous complex β-catenin, thus negatively regulating Wnt/β-catenin signaling. This decrease also resulted in the activation of proapoptotic pathways, regardless of GJIC formation [35]. These findings were observed in the late, and not the early, stage of colon carcinogenesis. In the premalignant stages, alterations in Wnt/β-catenin signaling are not common. In sporadic colon carcinogenesis in humans, mutations in the tumor suppressor adenomatous polyposis coli (APC) gene, which is a negative regulator of the Wnt/β-catenin pathway, are rare (0–11%) [28]. In a rat model of colon carcinogenesis induced by DMH, no ACF had *Apc* mutations, and only a minor part harbored *Ctnnb1* (β-catenin) mutations (7%). Mutations in *Apc* (~30%) also do not occur in most benign tumors [67,68]. In this current study, no nuclear staining for β-catenin in CRA was found, only a membranous and/or cytoplasmic pattern, indicating no nuclear translocation and Wnt/β-catenin pathway activation. In contrast, inactivating mutations in APC have been reported in up to 70% of sporadic CRC patients [69], and *Ctnnb1* mutations were found in ~40% of DMH-induced CRC in rats [70]. Thus, the absence of the effects of Cx43 deficiency on the earliest endpoint lesions (ACF and CRA) in mouse colon carcinogenesis may be explained by the increasing importance of Wnt/β-catenin pathway activation during the late stages this disease. Noteworthy, most clinical findings indicate that the absence/decreased expression of Cx43 is correlated to an advanced disease stage and the presence of metastasis [35,36,37]. The role of Cx43 deficiency in the CRA progression to CRC (malignant transformation) should also be considered. The in silico cohorts corroborated with these findings, as the downregulation of the Cx43 gene in CRA was not correlated to proliferation (MKI67), apoptosis (CASP3) markers, β-catenin, and other classically altered genes during colon carcinogenesis. Although the number of samples was low, clinical studies are usually performed on whole biopsy samples. Moreover, in silico and genome-wide in vivo investigations on the implications of Cx43 in ACF are encouraged. Although we found discrete effects of Cx43 deficiency on apoptosis in CRAs, further investigations should consider a recent mechanism described in vitro, showing that selenium nanoparticles promote the apoptosis of human glioblastoma cells by activating a Ca^2+^-dependent mechanism that also relies on Cx43 hemichannel activity [71].

## 5. Conclusions

Altogether, the findings of this animal study point to the discrete implication of Cx43 in the early stages of chemically induced colon carcinogenesis. As most of the available preclinical and clinical data indicate that Cx43 may function as a tumor suppressor in the late stages of colon carcinogenesis, these findings do not discard the potential clinical value of this protein.

## Data Availability

All relevant data are available in the manuscript, and additional information will be available upon reasonable request.

## Supplementary Materials

The following supporting information can be downloaded at: https://www.mdpi.com/article/10.3390/antiox11122368/s1: Figure S1: Representative agarose gel electrophoresis showing the results of PCR genotyping for wild-type (WT) or Connexin 43 heterozygous (Cx43^+/-^) mouse. Figure S2: In silico analysis of the correlation of the Cx43 gene (GJA1) with the Kras (KRAS) and p53 (TP53) mRNA levels in human (A) laser microdissected or (B) whole biopsy samples of normal mucosa/colon and colorectal adenoma (CRA). Figure S3: In silico analysis of the Cx43 gene (GJA1) and its correlation with the β-catenin (CTNNB1), Ki-67 (MKI67), caspase-3 (CASP3), Kras (KRAS), and p53 (TP53) mRNA levels in human (A) laser microdissected or (B) whole biopsy samples of normal mucosa/colon, colorectal adenoma (CRA), and colorectal carcinoma (CRC).
