# Correction: Santo et al. The Implications of Connexin 43 Deficiency During the Early Stages of Chemically Induced Mouse Colon Carcinogenesis. *Antioxidants* 2022, *11*, 2368

**DOI:** 10.3390/antiox15091066

**Published:** 2026-08-26

**Authors:** Sara Gomes Espírito Santo, Tereza Cristina da Silva, Mathieu Vinken, Bruno Cogliati, Luís Fernando Barbisan, Guilherme Ribeiro Romualdo

**Affiliations:** 1 Department of Pathology, Botucatu Medical School, São Paulo State University (UNESP), Botucatu 18618-687, São Paulo, Brazil; 2 School of Veterinary Medicine and Animal Science, University of São Paulo (USP), São Paulo 05508-270, São Paulo, Brazil; 3 Department of In Vitro Toxicology and Dermato-Cosmetology, Vrije Universiteit Brussel (VUB), 1090 Brussels, Belgium; 4 Department of Structural and Functional Biology, Botucatu Medical School, São Paulo State University (UNESP), Botucatu 18618-689, São Paulo, Brazil

In the original publication [1], there was a mistake in Figure 4A. Specifically, due to human error during figure preparation, the representative images of methylene blue-stained colon specimens shown for the Saline Control and Saline Cx43^+/−^ groups contain a partial overlap. The corrected Figure 4A appears below. 

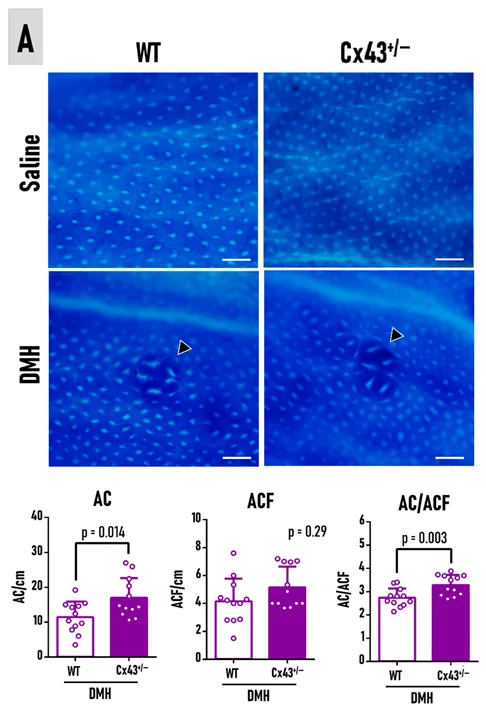



The authors state that the error does not affect the underlying raw data, quantitative analyses, statistical results, interpretation of the findings, or the conclusions of the study. This correction was approved by the Academic Editor. The original publication has also been updated.

## References

[1] Santo S.G.E., da Silva T.C., Vinken M., Cogliati B., Barbisan L.F., Romualdo G.R. (2022). The Implications of Connexin 43 Deficiency during the Early Stages of Chemically Induced Mouse Colon Carcinogenesis. Antioxidants.

