# Supplementary material for: The Implications of Connexin 43 Deficiency during the Early Stages of Chemically Induced Mouse Colon Carcinogenesis
Source: Antioxidants (Basel). 2022 Nov 30;11(12):2368. doi: 10.3390/antiox11122368 (PMC9774636; doi:10.3390/antiox11122368)
Supplement: Supplementary file 1 [file antioxidants-11-02368-s001.zip › antioxidants-2042958-supplementary.pdf]

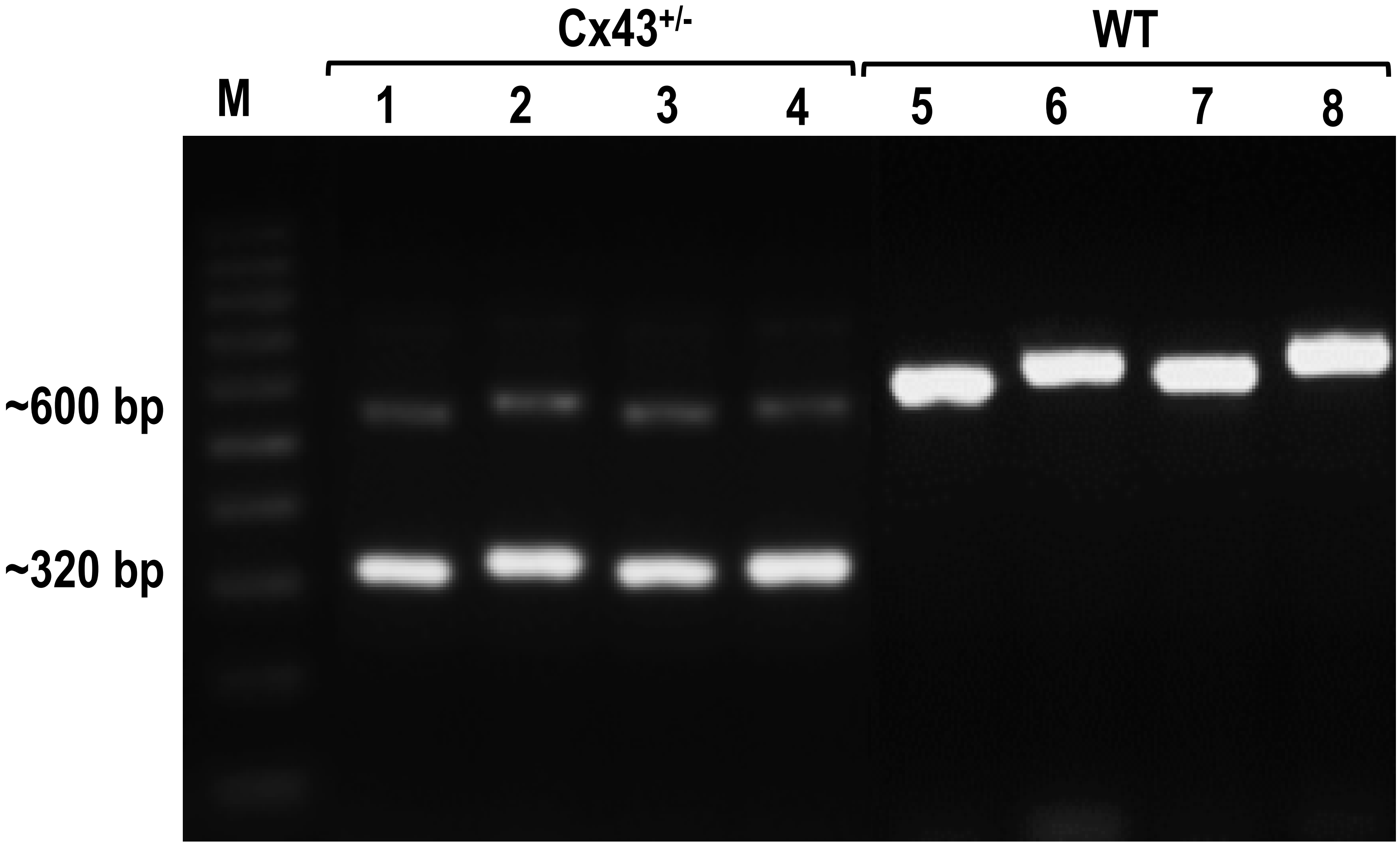

Supplementary Figure S1. Representative agarose gel electrophoresis showing results of PCR genotyping for wild-type (WT) or Connexin 43 heterozygous (Cx43$^{+/-}$) mouse. Lane M: Marker; Lanes 1-4: Cx43+/- mice, showing a normal allele at ~600 base pairs (bp), and mutant allele at ~320 base pairs (bp); Lanes 5-8: WT mice, showing both alleles at 600 bp.

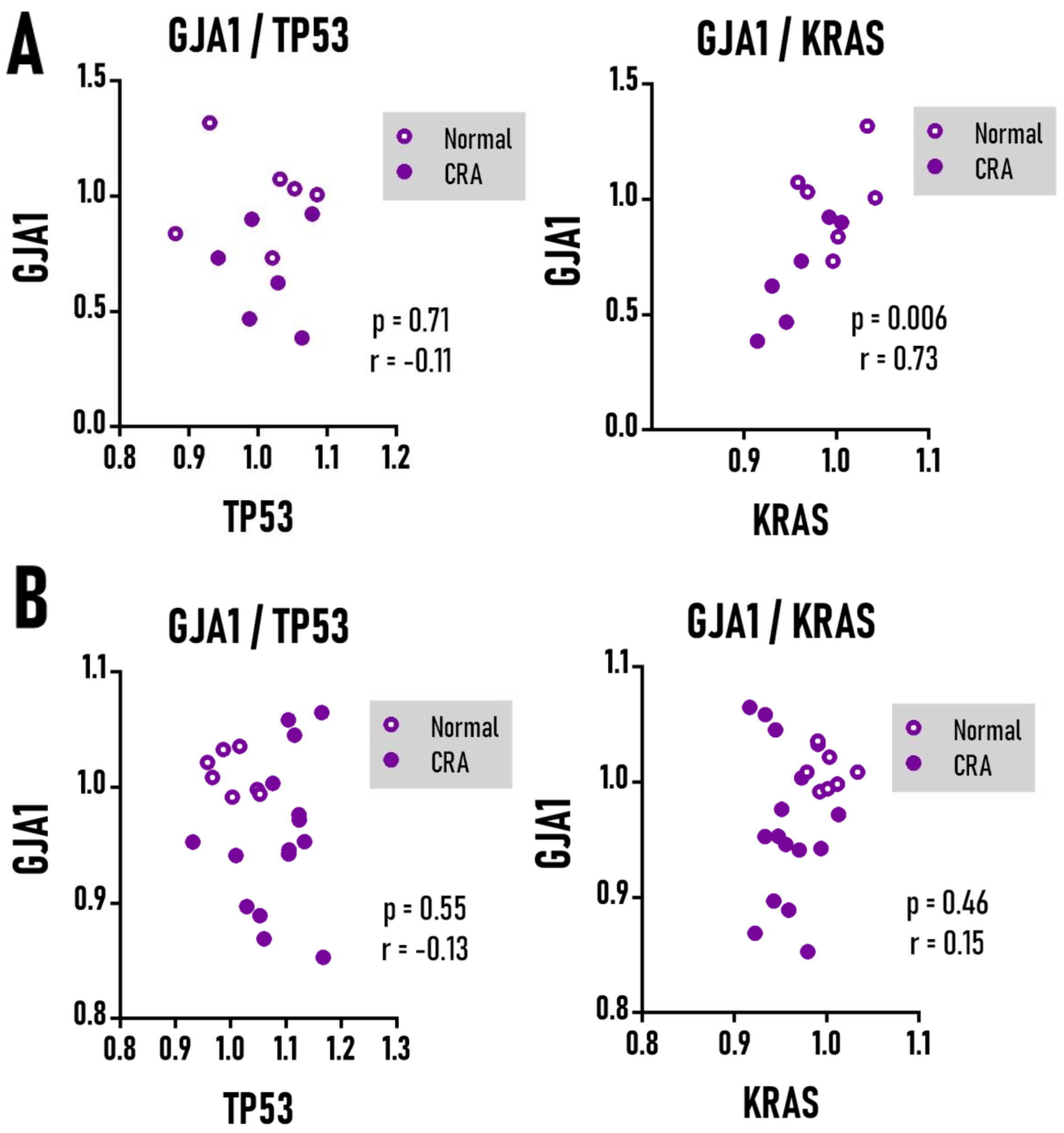

Supplementary Figure S2. *In silico* analysis of the correlation of Cx43 gene (GJA1) with Kras (KRAS), and p53 (TP53) mRNA levels in human (A) laser microdissected or (B) whole biopsy samples of normal mucosa/colon,and colorectal adenoma (CRA). A: human normal mucosa, CRA (n=6 for each class); B: normal colon (n=8), CRA (n=15) samples. Data are presented as mean ± standard deviation and datapoints. Data were analyzed one-way ANOVA and *post hoc* Tukey test or Pearson's correlation test ($p<0.05$).

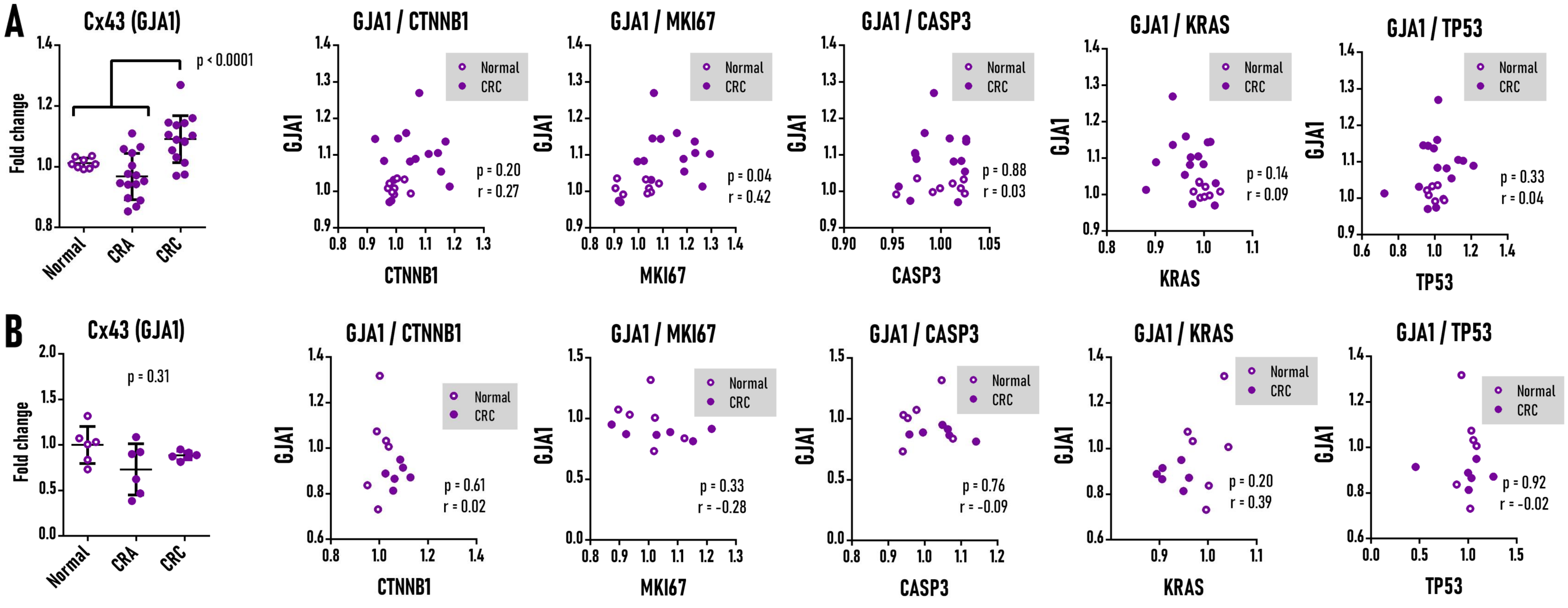

Supplementary Figure S3. *In silico* analysis of Cx43 gene (GJA1) and its correlation with β-catenin (CTNNB1), Ki-67 (MKI67), caspase-3 (CASP3), Kras (KRAS), and p53 (TP53) mRNA levels in human (A) laser microdissected or (B) whole biopsy samples of normal mucosa/colon, colorectal adenoma (CRA), and colorectal carcinoma (CRC). A: human normal mucosa, CRA and CRC samples (n=6 for each class); B: normal colon (n=8), CRA or CRC (n=15) samples. Data are presented as mean ± standard deviation and datapoints. Data were analyzed one-way ANOVA and *post hoc* Tukey test or Pearson's correlation test (p<0.05).
